# Selection and reliability of internal reference genes for quantitative PCR verification of transcriptomics during the differentiation process of porcine adult mesenchymal stem cells

**DOI:** 10.1186/scrt7

**Published:** 2010-03-30

**Authors:** Elisa Monaco, Massimo Bionaz, Alecsandra Sobreira de Lima, Walter L Hurley, Juan J Loor, Matthew B Wheeler

**Affiliations:** 1 Laboratory of Stem Cell Biology and Engineering, Department of Animal Sciences, University of Illinois at Urbana-Champaign, 1207 West Gregory Drive, Urbana, 61801, Illinois, USA; 2 Institute for Genomic Biology, University of Illinois at Urbana-Champaign, 1207 West Gregory Drive, Urbana, 61801, Illinois, USA; 3 Mammalian NutriPhysioGenomics, Department of Animal Sciences, University of Illinois at Urbana-Champaign, 1207 West Gregory Drive, Urbana, 61801, Illinois, USA

## Abstract

**Introduction:**

The objective of this study was to find highly reliable internal-control genes (ICGs) for normalization of qPCR data from porcine adult mesenchymal stem cells induced to differentiate toward adipogenic and osteogenic lineages.

**Methods:**

Stem cells were acquired from subcutaneous back fat and bone marrow of three castrated Yorkshire crossbred male pigs. Adipose and bone marrow-derived stem cells (ADSCs and BMSCs) were cultured *in vitro *with specific osteogenic or adipogenic differentiation medium for 4 weeks. Total RNA was extract for microarray (13,000 oligonucleotides) and qPCR analyses. Microarray data were used to uncover the most stably expressed genes (that is, potential ICGs). Co-regulation among potential ICGs was evaluated with Ingenuity Pathway Analysis. qPCR was performed on the non-coregulated ICGs candidates and on specific osteogenic (*COL1A1*) and adipogenic (*DBI*) genes. geNorm was used to uncover the most reliable ICGs by using qPCR data and the optimal number of ICGs to be used to calculate the normalization factor.

**Results:**

Microarray data analysis revealed 27 potential ICGs. Among those, 10 genes without known co-regulation were selected to perform qPCR. geNorm performed on qPCR data uncovered high stability in expression ratio among the selected ICGs. However, especially reliable normalization was obtained by geometric mean of *NSUN5*, *TIMM17B*, and *VPS4A*. The effect of normalization, assessed on specific osteogenic (*COL1A1*) and adipogenic (*DBI*) genes, was apparent for the adipogenic and less apparent for the osteogenic differentiation.

**Conclusions:**

The combination of microarray data and pairwise gene analysis allowed identification of novel and highly reliable ICGs for qPCR data normalization of adult porcine stem cells induced to differentiate to adipogenic and osteogenic lineages.

## Introduction

The quantity and accessibility of subcutaneous adipose tissue in humans makes it an attractive alternative to bone marrow as a source of adult stem cells for therapeutic purposes [1]. However, little is known about similarities and differences between adipose- (ADSCs) and bone marrow-(BMSCs) derived stem cells at the genetic level and during their differentiation into major mesenchymal cell types. Uncovering transcriptomic adaptations of porcine adult mesenchymal stem cells during *in vitro *differentiation toward a target tissue can provide crucial information for human adult stem cell therapeutic applications.

The two most common techniques currently used for gene-expression analysis are real-time RT PCR (qPCR) and microarray. qPCR is the method of choice for accurate mRNA expression analysis of selected genes, and it is used for verification of microarray results. Although qPCR can provide accurate estimates of mRNA abundance in experimental samples, the technique is subject to errors during the steps from RNA extraction to mRNA quantitative analysis [2]. The qPCR data must be normalized to take those errors into account. To date, the most frequently used method to normalize qPCR data is through the use of internal control genes (ICGs), sometimes referred to as housekeeping genes (HKGs) [3]. Housekeeping-gene expression should not vary in the cells under investigation, or in response to a treatment. However, the literature shows that HKGs expression, although occasionally constant in a given cell type or experimental condition, can vary considerably [4-6]. As a consequence, the presumed stability of expression of these genes must be validated under the particular experimental conditions being investigated. In many studies, traditional HKGs (such as *ACTB*, *GAPDH*, *HPRT*, β-*actin*) are used to normalize qPCR data without a proper evaluation of their presumed stability of expression [7-9]. The use of appropriate ICGs to normalize qPCR data is an absolute prerequisite for reliable results.

The choice of IGCs using traditional approaches has been based strictly on the absence of statistical effects of a treatment or physiologic state on qPCR data. This criterion is not sufficiently stringent for studies in which a treatment or physiologic adaptation results in a large transcriptomic modification, as in case of cells undergoing differentiation [10]. The protocols used for qPCR require use of the same amount of total RNA for RT of each sample; and in the case of a large change in expression of some transcripts, the relative mRNA abundance of other transcripts may appear concentrated or diluted [11]. Genes with stable expression (that is, the ideal ICGs), also may appear to have a significant change in expression. Those genes would be considered unreliable as ICGs, even though the observed modification in expression is an artifact of the consistency of amount of starting RNA in each reaction [11,12]. The use of pair-wise expression ratio-stability comparisons among multiple candidate genes [3] has been found to be an appropriate method to overcome these limitations. This method, developed by Vandesompele *et al. *[3] is based on the use of multiple control genes for accurate normalization of qPCR data. The procedure can be applied in many biologic contests because it takes into account large transcriptomic modification that can follow to a treatment or physiologic adaptation.

Differentiation of stem cells may be expected to result in significant changes in gene expression. Evaluation of expression of genes used as markers for differentiated cells requires identification and validation of appropriate ICGs before conclusions can be drawn relative to transcriptomic adaptations of the differentiation process. The objectives of this study were (a) to identify highly reliable ICGs for normalization of qPCR data for porcine ADSCs and BMSCs, each induced to differentiate toward the adipogenic and osteogenic lineages; and (b) to propose a protocol implemented from the method developed by Vandesompele *et al. *[3], which can be used in many, if not all, cell/tissue-type experiments using qPCR. In addition, we provided a list of genes that can be considered suitable candidate ICGs to test in any experiment using porcine mesenchymal stem cells.

## Materials and methods

### ADSCs and BMSCs isolation and culture *in vitro*

Subcutaneous back fat and bone marrow were acquired from three castrated Yorkshire crossbred male pigs, at ~6 months of age, under protocols approved by the University of Illinois Institutional Animal Care and Use Committee (IACUC). Pigs were killed at the University of Illinois Meat Science Laboratory abattoir. Details on tissue harvesting and stem cells primary culture *in vitro *are as previously reported [13]. In brief, subcutaneous back fat and bone marrow from femurs were harvested and processed in sterility to avoid contamination of the samples. They were minced with sterile scalpel blades and digested with 0.075% collagenase type I-A (Sigma Aldrich C2674, St. Louis, MO) at 37°C for 90 min. After digestion, cells were separated by centrifugation at 200 *g *for 10 min at room temperature. The buoyant cell fraction and supernatant were discarded, and 2 ml of red blood cell lysis buffer (Sigma R7757) was added to the pellet and gently mixed for 2 min. Subsequently, 20 ml of Dulbecco's phosphate-buffered saline (DPBS, Sigma D5773) containing 1% penicillin G-streptomycin (Sigma P3539) and 5.0 mg/L of amphotericin B (Sigma A9528) were added to the tubes that were centrifuged at 200 *g *for 5 min, at room temperature, to obtain a cell pellet that was then resuspended in culture medium. The culture medium used was high-glucose Dulbecco's modified Eagle's medium (DMEM; Sigma D5648) supplemented with 10% fetal bovine serum (FBS, BenchMark; Gemini Bio Products, West Sacramento, CA) plus 1% penicillin G-streptomycin and 5.0 mg/L of amphotericin B. Cells were counted and plated in 75-cm^2 ^Corning cell culture flasks at 7.5 × 10^5 ^cells for ADSCs and 1.6 × 10^6 ^cells for BMSCs in 15 ml of culture medium and incubated at 39°C and 5% CO_2 _(passage 0 cells). Medium was changed every other day until cells reached 80% confluence (day 10 of culture). To keep the cells at a sufficiently low density to stimulate further growth, they were trypsinized with 0.25% Trypsin (Sigma T4799), 0.04% EDTA (Sigma E6753), and replated at the density indicated earlier (passage 1 cells). At 80% confluence, cells were trypsinized again and frozen and stored in liquid N_2 _until further use.

### ADSCs and BMSCs osteogenic and adipogenic differentiation *in vitro*

Frozen ADSCs and BMSCs were thawed and plated (passage 2) at a density of 7.5 × 10^5 ^cells/75 cm^2^. Cells were cultured for 4 days, and then they were trypsinized, counted, and plated (passage 3) again for 4 days before induction of differentiation. To induce the differentiation of ADSCs and BMSCs into osteogenic or adipogenic cells *in vitro*, ADSCs and BMSCs were cultured with specific osteogenic or adipogenic differentiation medium. Osteogenic medium consisted of basic high-glucose DMEM supplemented with 100 nmol/L dexamethasone (Sigma D4902), 10 mmol/L β-glycerophosphate (Sigma G6376), 0.05 mmol/L ascorbic acid-2-phosphate (Sigma A4403), 10% FBS, 1% penicillin G-streptomycin, and 5.6 mg/L of amphotericin B. Adipogenic medium consisted of basic high-glucose DMEM supplemented with 1.0 μmol/L dexamethasone, 0.5 mmol/L isobutylmethylxanthine (IBMX, Sigma I5879), 10 μmol/L insulin (Sigma I9278), 200 μmol/L indomethacin (Sigma I7378), 10% FBS, 1% penicillin G-streptomycin, and 5.6 mg/L of amphotericin B. Cells were cultured in these media for 3 weeks, with media changed every 3 days.

### RNA Extraction, qPCR, and primer design and testing

At 0, 2, 7, and 21 days of differentiation (dd) cells were trypsinized, as described earlier and counted by hemocytometer. Total RNA was immediately isolated by using the RNeasy Mini Kit (Qiagen Sciences, Germantown, MD) according to the manufacturer's protocol. Any residual genomic DNA was eliminated by treatment with RNase-Free DNase Set (Qiagen). RNA concentration was measured with a NanoDrop ND-1000 spectrophotometer (NanoDrop Technologies, Wilmington, DE). A portion of the highly pure RNA (A260/A280 = 2.13 ± 0.05) was diluted to 100 mg/L by using DNase-RNase-free water before reverse transcription. Sufficient cDNA was prepared to run all selected genes. Each cDNA was synthesized by reverse transcription and used for qPCR, as described by Bionaz and Loor [11]. Each sample was run in triplicate along with a 6-point relative standard curve plus the nontemplate control. The fivefold dilution standard curve was made by using cDNA from an RNA pool of four swine tissues: liver, mammary gland, jejunum, and kidney. The amplification reactions were conducted in an ABI Prism 7900 HT SDS instrument (Applied Biosystems) as follows: 2 min at 50°C, 10 min at 95°C, 40 cycles of 15 s at 95°C, and 1 min at 60°C. The presence of a single PCR product was verified by the dissociation protocol by using incremental temperatures to 95°C for 15 seconds plus 65°C for 15 seconds. Data were analyzed with the 7900 HT Sequence Detection Systems software (version 2.2.3, Applied Biosystems). Primer design and evaluation were performed as previously described by Tramontana *et al. *[12].

### Selection and evaluation of ICGs

For the purpose of identifying potential ICGs, porcine microarray (13,000 oligonucleotides) data generated by a dye-swap reference design [10] were mined to uncover stable expressed genes during osteogenic and adipogenic differentiation *in vitro *of ADSCs and BMSCs. The protocol to select and evaluate ICGs by using microarray was as previously described [12].

The most stably expressed genes among all samples in the microarray data set were chosen by following these criteria: (a) only genes with median intensity ≥3 standard deviations above the median of the background were used; (b) filtered data were uploaded into GeneSpring GX7 software (Agilent Technologies) and normalized by using Lowess; (c) only genes with expression ≥100 relative fluorescent units (that is, medium-large mRNA abundance) were chosen; and (d) only genes with expression ratio ~1.0 between sample and reference (RNA mixture from porcine mammary gland, liver, kidney, and jejunum) and with complete annotation were selected.

### Gene-stability evaluation

To uncover reliable ICGs, we used geNorm software, which is based on a pairwise expression ratio stability comparison method [3]. geNorm measures the stability (*M*) of the ICGs- as the average pairwise expression ratio variation of qPCR data of a particular gene with all other control genes tested. Genes with the lowest *M *values have the most stable expression. geNorm also determines the optimal number of ICGs and calculates a normalization factor (NF) to be used for normalization [3]. It uses a pairwise variation analysis between the normalization factors NFn and NFn+1 (V) to determine the number of control genes required for accurate normalization. The pairwise comparison requires evaluation of multiple genes to select appropriate internal controls. Critical for the use of the pairwise comparison method in selecting appropriate ICGs is the absence of co-regulation (common upstream regulator factor(s) or direct regulation of transcript expression between gene products) among the genes tested [3]. Co-regulation was assessed by using Ingenuity Pathways Analysis (IPA; Ingenuity System). IPA is a web-based software that generates relations among human and rodent genes or proteins by using information from the published literature.

The evaluation of the appropriate number of ICGs to calculate the NF and the effect of normalization versus nonnormalization was evaluated by running qPCR for collagen type I alpha 1 (*COL1A1*) and diazepam-binding inhibitor (acyl-CoA-binding protein; *DBI*) which represented osteogenic and adipogenic gene markers, respectively.

### Statistical analysis

A Mixed model with repeated measures (SAS, release 9.0; SAS Inst. Inc., Cary, NC) was used to evaluate the effect of time and differentiation on raw (that is, nonnormalized) and normalized mRNA abundance. Spatial power was a suitable covariate structure based on Akaike's information criterion (closer to 0) for the model used. However, other covariate structures, such as autoregressive order 1 and compound symmetry, had an identical fitting. The model included the fixed effect of time (0, 2, 7, and 21 days), differentiation (osteogenic and adipogenic), cell type (ADSCs, BMSCs), and all interactions (Table 1). Pig (n = 3) was considered a random effect. Statistical correlation between qPCR data of ICGs with total RNA, number of cells, and concentration of total RNA/cell was performed by using the PROC CORR procedure of SAS.

**Table 1:** Statistical effect of each variable and combination of variables on 10 potential internal-control genes

Effect	BANF1	DAK	DPH3	GTF2H3	NSUN5	NUBP1	PRR3	SSU72	TIMM17b	VPS4A
Tissue	0.074	0.194	<.0001	0.817	0.279	0.718	0.144	<.0001	0.259	0.327
Differentiation	<.0001	0.008	0.006	0.001	0.889	0.041	<.0001	0.006	0.428	0.229
Day	<.0001	<.0001	<.0001	0.002	<.0001	<.0001	<.0001	0.001	<.0001	<.0001
Tissue* Differentiation	0.003	0.032	0.061	0.005	0.011	0.045	<.0001	0.008	0.026	0.006
Tissue* Day	<.0001	<.0001	<.0001	<.0001	<.0001	<.0001	<.0001	<.0001	<.0001	<.0001
Differentiation* Day	<.0001	0.022	0.140	0.000	0.754	0.004	<.0001	0.002	0.000	0.201
Tissue* Different* Day	0.271	0.374	0.033	0.086	0.395	0.271	0.009	0.097	0.503	0.323

## Results and Discussion

### ICGs selection and evaluation

The use of microarray data [7] allowed the identification of 27 stably expressed genes among >10,000 annotated transcripts (Table S1 in Additional file 1). The 27 genes were highly stable across all samples (ratio sample/reference = 0.99 ± 0.14) in at least 70 of 96 microarrays and represented a novel potential set of ICGs for qPCR analysis of porcine adult mesenchymal stem cells differentiated *in vitro*.

This preliminary screening allowed the identification of genes with similar mRNA abundance between samples (ADSCs and BMSCs) and the mixture of porcine tissues (reference in the microarray). They were genes with potentially stable expression, similar to those that are considered HKGs [14]. However, as previously suggested [11,12,15], the sole reliance on microarray data for evaluation of ICGs for qPCR normalization is inappropriate and should be considered only as a preliminary screening to identify genes with a potential for stable expression. The qPCR and array techniques differ in analytic procedure and sensitivity [16]. To overcome these differences and optimize the evaluation of ICGs for qPCR normalization, several tools have been developed, based on specific algorithms [3,17,18]. In particular, the geNorm software is a valuable tool to evaluate ICGs under diverse experimental conditions, and it is one of the most widely used softwares for evaluation of ICGs to date.

**Figure 1:**
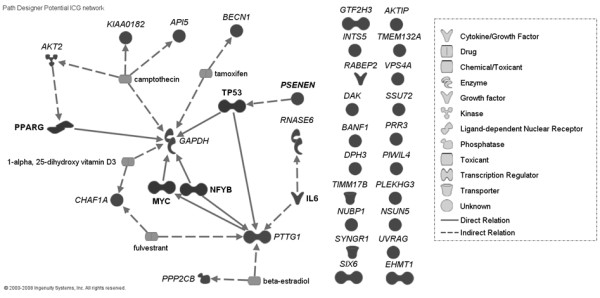
**Potential internal-control genes network**. Evaluation of potential co-regulation (that is, common upstream regulator(s), including endogenous chemical molecules) among the 27 genes with expression ratio between samples and reference close to 1 in a large microarray analysis.

The appropriate evaluation of ICGs by using geNorm requires the absence of co-regulation between the selected genes [3]. Co-regulated genes would be affected similarly under the same experimental conditions, which will bias the geNorm analysis (that is, ratio expression stability among two genes across samples). IPA was used to evaluate the presence of co-regulation among the 27 selected genes. This analysis allowed the identification of 20 genes without known co-regulation that were used for stability analysis (Figure 1). The genes that presented co-regulation were excluded from further analysis. Optimal primers were designed for and qPCR was performed on 10 of 20 genes without known co-regulation (Table S2 in Additional file 1). It was not possible to design appropriate primers for the remaining 10 genes without known co-regulation because of the absence of sequence availability or because of low primer-pair quality. The description of the 10 genes that were tested is reported in Table 2.

**Table 2:** Description of 10 potential internal-control genes

Gene	Description
*BANF1*	Barrier to autointegration factor 1
*DAK*	Dihydroxyacetone kinase 2 homologue (*S. cerevisiae*)
*DPH3*	KTI11 homologue (*S. cerevisiae*)
*GTF2H3*	Excision repair cross-complementing repair deficiency, complementation group 3
*NSUN5*	NOL1/NOP2/Sun domain family, member 5
*NUBP1*	Nucleotide-binding protein 1 (MinD homologue, *E. coli*)
*PRR3*	Proline-rich 3
*SSU72*	RNA polymerase II CTD phosphatase homologue (*S. cerevisiae*)
*TIMM17B*	Translocase of inner mitochondrial membrane 17 homologue B (yeast)
*VPS4A*	Vacuolar protein sorting 4 homologue A (*S. cerevisiae*)
*COL1A1*	Collagen, type I, alpha 1
*DBI*	Diazepam-binding inhibitor (acyl-coenzyme A-binding protein)

### ICGs expression pattern

**Figure 2:**
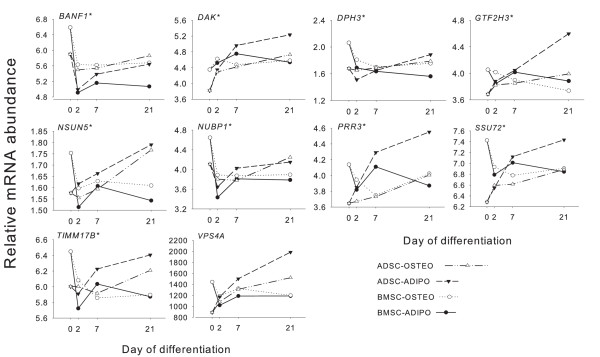
**Relative mRNA abundance of 10 potential internal-control genes**. Pattern of relative mRNA abundance of 10 potential internal-control genes tested in ADSCs and BMSCs during osteogenic and adipogenic differentiation *in vitro*. These data are raw data (that is, not normalized).

Large and significant time, time × tissue, and tissue × differentiation effects were observed in measured mRNA for all genes tested (Figure 2, Table 1). In general, a decrease in expression pattern was noted of almost all the 10 genes from dd 0 to dd 2, followed by an increased expression (Figure 2). The statistical effects of time, time × tissue, and tissue × differentiation on the potential ICGs did not exclude the possibility of a good fit for these genes as ICGs. In this regard, it has been demonstrated that the absence of statistical effect on the potential ICGs is not an essential condition to consider them reliable, and, in certain cases, the use of this as the only criterion for selecting appropriate ICGs can be a serious limitation [11,12]. Moreover, it has been shown that when a physiologic adaptation is featured by a large increase in mRNA synthesis of some abundantly expressed genes, the expression patterns of stably expressed genes appear downregulated because of a dilution effect, which is an artifact of the qPCR protocol [11]. In this case, a significant inverse correlation between qPCR data of reliable ICGs and amount of total RNA/tissue was observed [11]. However, in the present study, no apparent overall correlation was observed between the qPCR data of tested ICGs and the amount of RNA/cell (Table 3). Conversely, the negative correlation between number of cells and nanograms of RNA/cell (Table 3) suggests a decreased efficiency of RNA extraction with increasing cell number. Thus, correlations between ICGs and total RNA cannot be used to draw conclusions about a possible dilution (or concentration) effect of genes with stable expression (that is, ICGs) [11].

**Table 3:** Correlation between number of cell, RNA, and raw qPCR data of internal control genes tested.

	# cell	Tot RNA	*BANF1*	*DAK*	*DPH3*	*GTF2H3*	*NSUN5*	*NUBP1*	*PRR3*	*SSU72*	*TIMM17B*	*VPS4A*
# cell			-0.05	-0.08	-0.15	-0.26	-0.20	-0.18	-0.31	-0.11	***-0.35***	-0.11
Total RNA	***0.55***		0.07	-0.21	0.00	-0.21	-0.10	-0.08	***-0.42***	-0.09	-0.20	-0.01
ng RNA/cell	***-0.42***	***0.43***	0.06	0.11	0.20	0.14	0.20	0.09	0.06	0.11	0.28	0.27

In ***italicized bold***, correlation with *P *< 0.05.

### Considerations for selecting optimal number of ICGs

**Figure 3:**
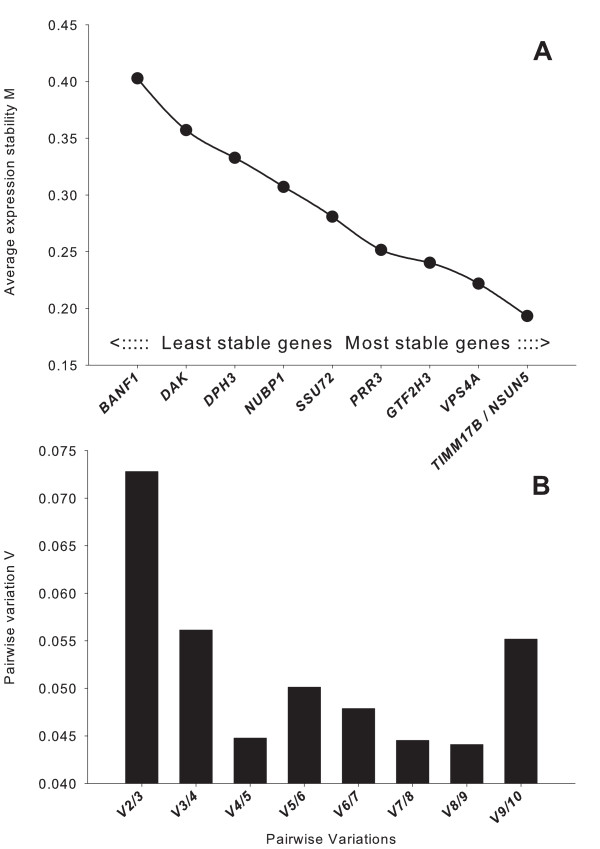
**Most-stable genes by geNorm and calculation of the optimal number of internal control genes**. **(a) **geNorm results for the average expression ratio stability (*M*) of potential internal-control genes calculated by pairwise comparison. **(b) **determination of the optimal internal-control genes number for normalization (lowest *M*).

**Figure 4:**
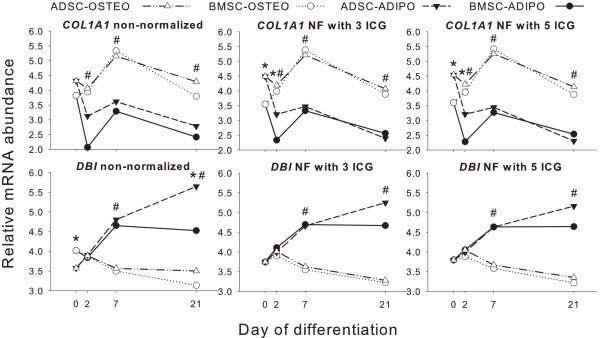
**Relative mRNA abundance of *COL1A1 *and *DBI *nonnormalized and normalized with three or five internal control genes**. Pattern of *COL1A1 *and *DBI *expression before and after normalization by using a NF calculated with three or five most-stable internal control genes from geNorm analysis (see Figure 3). * *P *< 0.05 for Tissue × Day interaction; ^#^*P *< 0.05 for Differentiation × Day interaction at each time point. See Table S3 for overall statistical results.

geNorm uncovered *TIMM17B *and *NSUN5 *as the genes with the most stable expression ratio (that is, lowest *M*) among evaluated ICGs calculated by pairwise comparison (Figure 3). As suggested, a minimum of three ICGs should be used to calculate the NF [3]. However, geNorm analysis revealed nine ICGs as the optimal number to be used for NF calculation (pairwise variation V = 0.044). The use of eight or five genes also resulted in a high reliability (V = 0.045); in addition, the V value of three ICGs was particularly low (V = 0.073). Comparing normalized expression of *DBI *and *COL1A1 *by using the NF calculated by three versus five most-stable ICGs (Figure 4) confirmed that no apparent difference existed in normalized qPCR results by including the two additional ICGs to calculate NF. The final number of ICGs to be used to calculate the NF often is a tradeoff between practical considerations and accuracy [3]. Including an increasing number of genes when the NF does not significantly change does not add to the value of the normalization analyses [3]. Based on the results of gene stability, and on the limit of V < 0.1, as previously suggested to be acceptable [11], we elected to use the three most-stable ICGs (*NSUN5, TIMM17B*, and *VPS4A*) to calculate the NF for normalization of qPCR data of porcine ADSCs and BMSCs induced to differentiate toward the adipogenic and osteogenic lineages. *NSUN5 *has not been characterized in the pig genome. In humans, this gene encodes a protein with similarity to p120 (NOL1) a 120-kDa proliferation-associated nucleolar antigen that is a member of an evolutionarily conserved protein family. It is expressed ubiquitously, with higher expression in heart, placenta, and skeletal muscle. *NSUN5 *is thought to function as an *S*-adenosyl-L-methionine-dependent methyl-transferase whose absence may be associated with the pathogenesis of William syndrome [19]. *TIMM17B *is a gene localized on the X chromosome that encodes for a translocase present on the inner mitochondrial membrane in the human [20]. Translocases in the mitochondrial inner and outer membranes mediate translocation of nuclear-encoded mitochondrial proteins. The location of the gene on the X-chromosome may question how gender may affect the reliability of this gene as an ICG. In this study, all the animals used were males, but because it has been demonstrated that, in females, one of the two X chromosomes is mainly silenced [21], we would expect similar reliability of this gene as an ICG for both genders. The *VPS4A *gene in humans encodes for the AAA protein family (ATPases associated with diverse cellular activities), and it is the homologue of the yeast *VPS4 *protein that is involved in the transport of proteins out of a prevacuolar/endosomal compartment. In a 1999 study, analysis of the promoter region of the gene revealed features common to HKGs [22].

The effect of normalization on qPCR data is highlighted by *COL1A1 *and *DBI *expression patterns before and after normalization (Figure 4, Table S3 for overall statistical results). For *COL1A1*, the Tissue × Day interaction was not significant when the gene was not normalized, but normalization with three or five ICGs gave a significant overall Tissue × Day interaction (Table S3); differences were observed at 0 and 2 days. Similarly, overall Tissue × Day interaction of *DBI *was significant before normalization, whereas after normalization, using three or five ICGs was not significant (Table S3), particularly for 0 days.

## Conclusions

Accurate normalization of qPCR data is an unavoidable step for obtaining reliable results. Many published studies dealing with the use of qPCR, however, do not report or follow a proper method to test for suitable ICGs. In this study, we indentified highly reliable ICGs for qPCR normalization data of porcine ADSCs and BMSCs. We also propose a protocol to select ICGs that can be used in many other cell/tissue-type experiments. The traditional criterion to select appropriate ICGs by using absence of time and/or treatment effect on qPCR data, would have rejected our candidate ICGs as inappropriate because of observed statistical effects on their expression. However, the use of IPA to select genes without known co-regulation, and the pairwise comparison method (geNorm), uncovered a very high stability in expression ratio among selected ICGs. This indicates that the relative mRNA abundance of those genes is similarly subjected to the errors before (for example, dilution/concentration effect) and during the qPCR procedure (from RNA extraction to qPCR analysis). The robustness of such an approach relies on the fact that the purpose for normalization of qPCR data is to correct for those errors. In addition, although the use of nine ICGs resulted in the optimal (that is, most stable) NF, the use of NF calculated by the geometric mean of *NSUN5*, *TIMM17B*, and *VPS4A *resulted in IGCs that are highly reliable for normalization of qPCR data from porcine adult mesenchymal stem cells induced to differentiate toward adipogenic and osteogenic lineages. Moreover, because it has been demonstrated that the expression patterns of commonly used ICGs can vary widely in different experimental conditions [23,24], the suitability of the ICGs uncovered in this study must be validated (for instance, by the simple use of geNorm), as the experimental conditions differ from those reported here. In this regard, the present work provides a large set of genes (Table S1 in Additional file 1) that can be used as potential ICGs, to be tested in similar experimental conditions in laboratories where the microarray data are not readily available.

## Abbreviations

ADSCs: adipose-derived stem cells; BMSCs: bone marrow-derived stem cells; DMEM: Dulbecco's Modified Eagle's Medium; DPBS: Dulbecco's phosphate-buffered saline; FBS: fetal bovine serum; HKGs: housekeeping genes; ICGs: internal control genes; IPA: ingenuity pathways analysis; NF: normalization factor.

Gene abbreviations are featured in Table 2.

## Competing interests

The authors declare that they have no competing interests.

## Authors' contributions

EM carried out the conception and design, collection and/or assembly of data, data analysis and interpretation, and manuscript writing. MB carried out the conception and design, data analysis and interpretation, and manuscript writing. ASL carried out the collection and/or assembly of data and data analysis. WLH carried out data analysis and interpretation and manuscript writing. JJL provided gene-expression and bioinformatics software and swine oligonucleotide microarray annotation. MBW carried out the conception and design, financial support, data analysis and interpretation, manuscript writing, and final approval of manuscript. All authors read and approved the final manuscript.

## Supplementary Material

Additional file 1 **Tables S1, S2 and S3**. **Table S1**: The ratio sample/reference and relative fluorescent units (RFUs) of the 27 selected genes with the most stable expression in 70 of 96 porcine microarray data from ADSCs and BMSCs induced to differentiate toward the osteogenic and adipogenic lineages. Annotation was based on similarity searches by using BLASTN and TBLASTX against human, mouse, and porcine UniGene databases, and the human genome. **Table S2: **The GenBank accession number, gene symbol, hybridization position, primer sequence, and amplicon size of primers used to analyze gene expression by qPCR. **Table S3: **The statistical effect of each variable and combination of variables on specific adipogenic (*DBI*) and osteogenic (*COL1A1*) genes nonnormalized (*NN*), normalized with three or five internal-control genes (ICGs). Click here for file
