# Supplementary material for: Selection and reliability of internal reference genes for quantitative PCR verification of transcriptomics during the differentiation process of porcine adult mesenchymal stem cells
Source: Stem Cell Res Ther. 2010 Mar 30;1(1):7. doi: 10.1186/scrt7 (PMC3226301; doi:10.1186/scrt7)
Supplement: Additional file 1 — Tables S1, S2 and S3. Table S1: The ratio sample/reference and relative fluorescent units (RFUs) of the 27 selected genes with the most stable expression in 70 of 96 porcine microarray data from ADSCs and BMSCs induced to differentiate toward the osteogenic and adipogenic lineages. Annotation was based on similarity searches by using BLASTN and TBLASTX against human, mouse, and porcine UniGene databases, and the human genome. Table S2: The GenBank accession number, gene symbol, hybridization position, primer sequence, and amplicon size of primers used to analyze gene expression by qPCR. Table S3: The statistical effect of each variable and combination of variables on specific adipogenic (DBI) and osteogenic (COL1A1) genes nonnormalized (NN), normalized with three or five internal-control genes (ICGs). [file scrt7-S1.DOC]

**ADDITIONAL FILES (Monaco et al., 2009)**

TABLE S1. Ratio sample/reference and relative fluorescent units (RFU) of the 27 selected genes with the most stable expression in 70 out of 96 porcine microarray data from ADSC and BMSC induced to differentiate toward the osteogenic and adipogenic lineages. Annotation was based on similarity searches using BLASTN and TBLASTX against human, mouse, and porcine UniGene databases, and the human genome.

| **Gene** | **Accession #** | **Description** | **Sample/reference** | | | **RFU intensity** | | | |
| --- | --- | --- | --- | --- | --- | --- | --- | --- | --- |
|  |  |  | ***Average*** | ***SD**** | ***CV^*^*** | ***Average*** | ***SD*** | ***Min*** | ***Max*** |
| AKT2 | NM_001626.3 | v-akt murine thymoma viral oncogene homolog 2 | 0.96 | 0.12 | 13.0 | 483 | 222 | 142 | 2565 |
| AKTIP | NM_001012398 | AKT interacting protein | 0.93 | 0.14 | 14.7 | 798 | 552 | 242 | 3589 |
| API5 | NM_006595.2 | apoptosis inhibitor 5 | 0.93 | 0.14 | 14.6 | 384 | 182 | 262 | 9585 |
| BANF1 | NM_003860.2 | barrier to autointegration factor 1 | 1.03 | 0.17 | 16.1 | 603 | 580 | 103 | 330 |
| BECN1 | NM_003766.2 | beclin 1 (coiled-coil, myosin-like BCL2 interacting protein) | 1.05 | 0.11 | 10.8 | 513 | 191 | 196 | 894 |
| CHAF1A | NM_005483.2 | chromatin assembly factor 1, subunit A (p150) | 0.96 | 0.12 | 12.1 | 3125 | 1835 | 127 | 2488 |
| DAK | NM_015533.3 | dihydroxyacetone kinase 2 homolog (S. cerevisiae) | 1.01 | 0.24 | 23.5 | 660 | 471 | 139 | 1853 |
| DPH3 | NM_206831.1 | DPH3, KTI11 homolog (S. cerevisiae) | 1.05 | 0.12 | 11.5 | 3200 | 2734 | 122 | 3486 |
| EHMT1 | NM_024757.3 | euchromatic histone-lysine N-methyltransferase 1 | 0.94 | 0.12 | 12.8 | 3142 | 2570 | 288 | 2902 |
| FLJ12529 | NM_024811.2 | pre-mRNA cleavage factor I, 59 kDa subunit | 1.03 | 0.17 | 16.7 | 1886 | 1514 | 223 | 8498 |
| INTS5 | NM_030628.1 | integrator complex subunit 5 | 1.02 | 0.15 | 14.6 | 463 | 339 | 109 | 1164 |
| KIAA0182 | NM_014615.1 | KIAA0182 | 1.05 | 0.10 | 9.7 | 350 | 107 | 430 | 2699 |
| NSUN5 | NM_148956.1 | NOL1/NOP2/Sun domain family, member 5 | 1.06 | 0.12 | 11.5 | 895 | 624 | 112 | 3993 |
| PIWIL4 | NM_152431.1 | piwi-like 4 (Drosophila) | 1.07 | 0.14 | 13.1 | 194 | 63 | 105 | 1152 |
| PLEKHG3 | NM_015549.1 | pleckstrin homology domain containing, family G (with RhoGef domain) member 3 | 0.91 | 0.13 | 13.8 | 1122 | 933 | 486 | 4150 |
| PRR3 | NM_025263.2 | proline rich 3 | 0.94 | 0.13 | 14.1 | 2319 | 1948 | 151 | 4862 |
| PSENEN | NM_172341.1 | presenilin enhancer 2 homolog (C. elegans) | 1.04 | 0.13 | 12.1 | 440 | 228 | 123 | 3451 |
| PTTG1 | NM_004219.2 | pituitary tumor-transforming 1 | 0.98 | 0.14 | 14.0 | 1247 | 556 | 109 | 946 |
| RABEP2 | NM_024816.2 | rabaptin, RAB GTPase binding effector protein 2 | 0.99 | 0.12 | 12.1 | 1870 | 989 | 696 | 4984 |
| RNASE6 | NM_005615.4 | ribonuclease, RNase A family, k6 | 1.02 | 0.14 | 13.4 | 1347 | 1076 | 145 | 588.5 |
| SIX6 | NM_007374.1 | SIX homeobox 6 | 0.92 | 0.12 | 12.8 | 2495 | 1057 | 201 | 7391 |
| SSU72 | NM_014188.2 | SSU72 RNA polymerase II CTD phosphatase homolog | 0.97 | 0.15 | 15.8 | 2953 | 2649 | 105 | 850.8 |
| SYNGR1 | NM_145731.3 | synaptogyrin 1 | 0.92 | 0.14 | 15.5 | 916 | 800 | 917 | 8663 |
| TIMM17B | NM_005834.1 | translocase of inner mitochondrial membrane 17 homolog B | 0.92 | 0.13 | 13.6 | 545 | 326 | 200 | 9465 |
| TMEM132A | NM_017870.3 | transmembrane protein 132A | 1.01 | 0.12 | 11.6 | 846 | 502 | 295 | 9564 |
| UVRAG | BE703491 | UV radiation resistance associated gene | 1.04 | 0.12 | 11.7 | 1035 | 676 | 166 | 1538 |
| VPS4A | NM_013245.2 | vacuolar protein sorting 4 homolog A (S. cerevisiae) | 0.93 | 0.11 | 12.2 | 1017 | 664 | 140 | 2110 |

^*^SD = standard deviation; CV = coefficient of variation

TABLE S2. GenBank accession number, gene symbol, hybridization position, primer sequence, and amplicon size of primers used to analyze gene expression by qPCR.

| **Accession no.** | **Gene** | **Primer^1^** | **Primer^2^ (5’-3’)** | **Amplicon size (bp)** |
| --- | --- | --- | --- | --- |
| Ak236683 | *BANF1* | F. 1094 | CACCATGTCACCTTTTTTGGAA | 107 |
|  |  | R. 1200 | ACCAGGAATTCACTCAGAAAAGCT |  |
| AK 237051 | *DAK* | F. 1655 | CCTGCCCAGCTACTCTCCAA | 73 |
|  |  | R. 1727 | CATAGAGTGCCCCGGATGA |  |
| Ak233502 | *DPH3* | F. 346 | GCTATCAGCAAGAAATTGAATCCA | 100 |
|  |  | R. 445 | CAGAGCGCTTCTTCTCCTACTTTAA |  |
| AK240475 | *GTF2H3* | F. 1222 | CATGCGAGACAGCCTTTAAGATT | 110 |
|  |  | R. 1331 | CAGCTCTACATGATGGAGAAAAAATT |  |
| Ak236125 | *NSUN5* | F. 710 | CCCCGGGTTCCCATGT | 90 |
|  |  | R. 799 | AGATCTTCCCTTGGTTCTTGAGAA |  |
| AK233504 | *NUBP1* | F. 416 | ACTGGAGGGAGAACAGGTTCAC | 105 |
|  |  | R. 520 | TCAGGACTGCTGAGCAAGAAAC |  |
| Ak240011 | *PRR3* | F. 718 | GCTGCCTCCTGGATCCTCTT | 113 |
|  |  | R. 830 | CTCAGGAACCAGGGCGATAC |  |
| AK240431 | *SSU72* | F. 334 | AACTCTACACGCAGAATGGCATT | 102 |
|  |  | R. 435 | TCAGGTCAAACAGGTCTTTGCA |  |
| AY609805 | *TIMM17B* | F. 143 | GAATCCGGCACCGATTGAG | 73 |
|  |  | R. 215 | GCGAAGCTACCTCCAATCTGA |  |
| AK239378 | VPS4A | F. 396 | GAGAGCAAGGGCAGTGATAGTGA | 79 |
|  |  | R. 474 | CGGCACCCATCAGCTGTT |  |
| AF201723 | *COL1A1* | F. 85 | AGAAGAAGACATCCCACCAGTCA | 105 |
|  |  | R. 189 | CCGTTGTCGCAGACACAGAT |  |
| NM_214119.1 | *DBI* | F. 49 | CAGGCGGAGTTTGAGAAAGCT | 100 |
|  |  | R. 148 | TCGCTTGTTTGTAGTGGCTGTAG |  |

^1^ Primer direction (F = forward; R = reverse) and hybridization position for each primer **(5’-3’).**

^2^ Exon-exon junctions in primer sequences are underlined.

**TABLE S3** Statistical effect of each variable and combination of variables on specific adipogenic (*DBI*) and osteogenic (*COL1A1*) genes non-normalized (*NN*), normalized with 3 or 5 internal control genes (ICG).

| Effect |  | *DBI* | | |  | *COL1A1* | | |
| --- | --- | --- | --- | --- | --- | --- | --- | --- |
|  |  | *NN^1^* | 3 ICG^2^ | 5 ICG^3^ |  | *NN* | 3 ICG | 5 ICG |
| Tissue |  | 0.0694 | 0.1404 | 0.0823 |  | 0.0003 | 0.0004 | 0.0001 |
| Differentiation |  | <.0001 | <.0001 | <.0001 |  | <.0001 | <.0001 | <.0001 |
| Day |  | <.0001 | <.0001 | 0.0012 |  | <.0001 | <.0001 | <.0001 |
| Tissue*Different |  | 0.0712 | 0.6847 | 0.8884 |  | 0.0964 | 0.4051 | 0.4817 |
| Tissue*Day |  | <.0001 | 0.1482 | 0.2064 |  | 0.2609 | 0.002 | 0.0014 |
| Different*Day |  | <.0001 | <.0001 | <.0001 |  | <.0001 | <.0001 | <.0001 |
| Tissue*Diff*Day |  | 0.1122 | 0.1135 | 0.3185 |  | 0.2094 | 0.2826 | 0.1591 |

^1^ Non-normalized

^2^ Normalized with 3 internal control genes

^3^ Normalized with 5 internal control genes
